# Phylogenetic constrains on *Polyporus umbellatus*-*Armillaria* associations

**DOI:** 10.1038/s41598-017-04578-9

**Published:** 2017-06-26

**Authors:** Xiaoke Xing, Jinxin Men, Shunxing Guo

**Affiliations:** Institute of Medicinal Plant Development, Chinese Academy of Medical Sciences and Peking Union Medical College, Beijing, 100193 China

## Abstract

It has been well established that some *Armillaria* species are symbionts of *Polyporus umbellatus*, However, little is known about the evolutionary history of *P. umbellatus*-*Armillaria* associations. In this research, we used an analysis based on the strength of the phylogenetic signal to investigate *P. umbellatus*-*Armillaria* associations in 57 sclerotial samples across 11 provinces of China. We isolated *Armillaria* strains from the invasion cavity inside the sclerotia of *P. umbellatus* and then phylogenetically analyzed these *Armillaria* isolates. We also tested the effect of *P. umbellatus* and *Armillaria* phylogenies on the *P. umbellatus*-*Armillaria* associations. We isolated forty-seven *Armillaria* strains from 26 *P. umbellatus* sclerotial samples. All *Armillaria* isolates were classified into the 5 phylogenetic lineages found in China except for one singleton. Among the 5 phylogenetic lineages, one lineage (lineage 8) was recognized by delimitation of an uncertain phylogenetic lineage in previous study. Results of simple Mantel test implied that phylogenetically related *P. umbellatus* populations tend to interact with phylogenetically related *Armillaria* species. Phylogenetic network analyses revealed that the interaction between *P. umbellatus* and *Armillaria* is significantly influenced by the phylogenetic relationships between the *Armillaria* species.

## Introduction

*Polyporus umbellatus* (Pers.) Fries, belonging to Polyporaceae, is a widespread medicinal fungus which mainly distributed in China, Japan and other temperate regions of the Northern Hemisphere^1^. The dried sclerotia of *P. umbellatus* has been used as herbal medicine in China for more than 2000 years in China to cure edema and promote diuretic processes^2^. In recent years, a polysaccharide from *P. umbellatus* sclerotia was shown to promote anti-tumor and immunomodulating activities^3, 4^. At present, the supply of *P. umbellatus* for medicinal purposes is mainly dependent on wild collection. Increasing commercial demands and less effective protection have led to excessive harvests and a dramatic decline of wild *P. umbellatus* resources in China^5^.

*Armillaria* (Fr.) Staude (Physalacriaceae, Agaricales, Basidiomycota) is one of the most important of the macrofungi with world-wide distribution. Some species are important root rot pathogens of forest plants^6^, and some species exhibit high nutritional and medicinal value^7^.In early years, the taxonomy of *Armillaria* was established mainly via mating tests. At present, approximately 40 biological species have been reported with global range^8^. Among them, less than 30 species have been named, while the others are still called “biological species”. In China, 16 Chinese Biological species (CBS A to CBS P) of the *Armillaria* have been defined based on mating tests^9, 10^. However, due to the limits of mating tests, some ambiguous and confused biological species still need be further revised via modern molecular techniques, especially DNA-based analysis, e.g. rDNA ITS, IGS, β-tubulin, elongation factor-1 alpha (EF-1α), and combined multilocus sequence analysis. Coetzee *et al*.^11^ phylogenetically analyzed CBS, and elucidated four main phylogenetic clusters, i.e., *A. ostoyae*, *A. gallica*, *A.tabescens*, and *A. mellea* clusters. However, the relationship between the CBS and phylogenetic clades is still unclear and most of the CBS remain unnamed. Recently, Guo *et al*.^12^ revealed fifteen phylogenetic lineages of *Armillaria* from China, of which seven were newly discovered and two were recorded for the first time in China. Their work effectively established the link between the CBS and the phylogenetic lineages.

Some *Armillaria* species have been shown to be symbionts of *P. umbellatus*. It has been established that growth of *P. umbellatus* sclerotia is mainly dependent on *Armillaria* spp. to supply needed nutrition^13^. Based on this result, there have been attempts to produce *P. umbellatus* sclerotia in some provinces of China, via dual culture of small sclerotia of *P. umbellatus* with twigs or sticks which had been previously infected by rhizomorph of *Armillaria* spp.^14^. However, this kind of cultivation has experienced problems with both the quality of the sclerotia and production efficiency due to lack of information regarding the species and ecological characteristics of the *Arimillaria* used. Although *Armillaia* is an important factor that determines the efficiency and mass production of cultivated *P. umbellatus*, there have been few studies on the association of *Armillaria* species with *P. umbellatus*. In most of the books and articles related to *P. umbellatus*, the *Armillaria* were described as *A. mellea* or *Armillaria* spp. Kikuchi & Yamaji^15^ implied that *Armillaria* species which associated with *P. umbellatus* were some unidentified *Armillaria* biological species closely related to *A. sinapina*, *A. calvescens*, *A. gallica*, *A. cepistipes*, and *A. nabsnona*. However, due to their small sampling size (three *P. umbellatus* sclerotial samples from Japan and China, respectively), this finding requires further verification. In addition to the ambiguous *Armillaria* spp. with which *P. umbellatus* associates, *P. umbellatus* also exhibits high intraspecific diversification^16^. This raises questions of whether there is a phylogenetic signal in the mutual selection between *P. umbellatus* and *Armillaria* during the long-term evolutionary process, i.e. whether more closely related *P. umbellatus* populations tend to form symbiotic associations with more closely related *Armillaria* species.

To develop a better understanding of the evolutionary history of *P. umbellatus* and *Armillaria* associations, we collected 57 sclerotial samples of *P. umbellatus* from 11 provinces in China, and we successfully isolated 47 *Armillaria* strains. The aim of this paper is to elucidate: (1) the *Armillaria* species that associate with *P. umbellatus*, (2) whether closely related *P. umbellatus* populations associate with closely related *Armillaria* species, and (3) whether the phylogenetic signal significantly drives the interaction between *P. umbellatus* and *Armillaria*?

## Results

### *Armillaria* species associated with *P. umbellatus*

**Figure 1:**
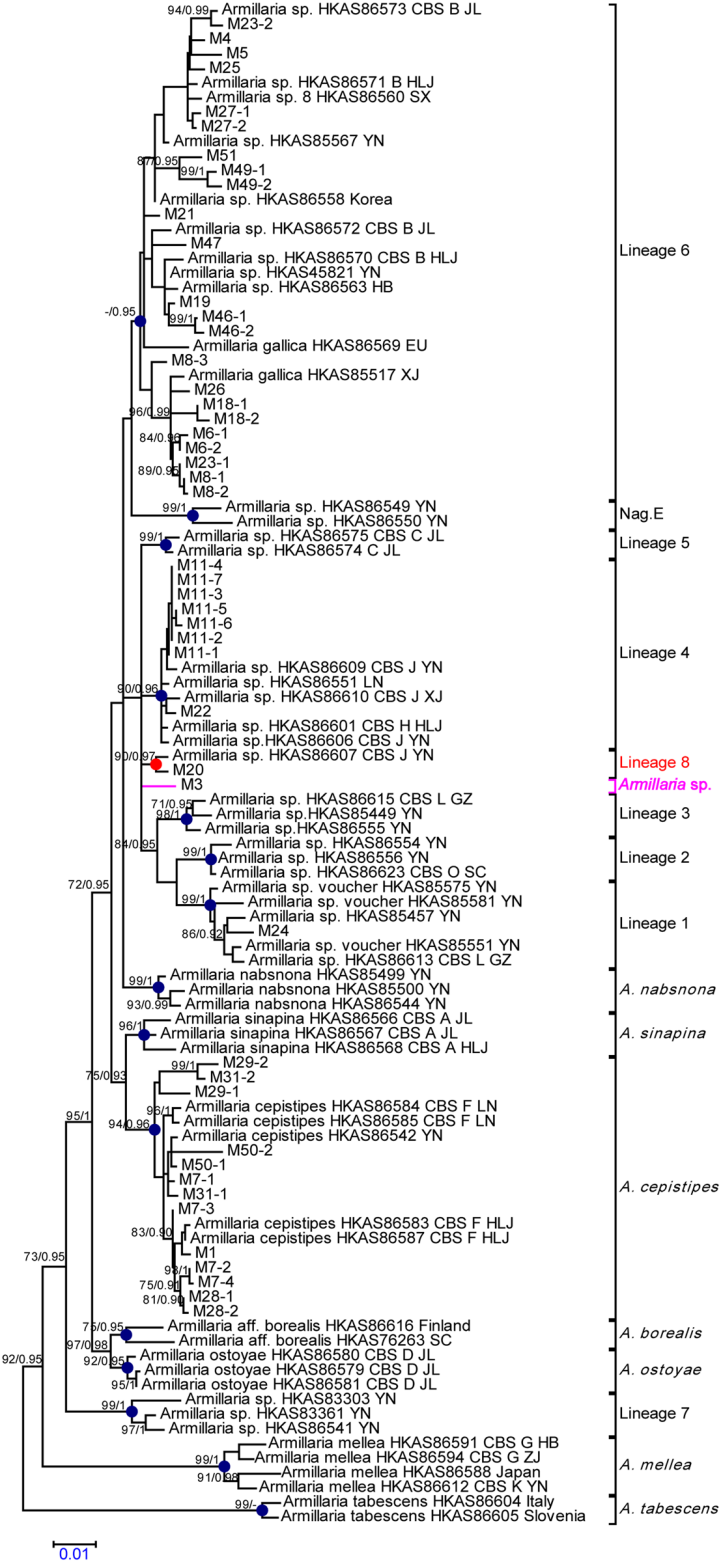
Phylogenetic tree generated from the three-locus (ITS, EF1-α and β-tubulin) data set. The blue labels on the nodes of the phylogram indicate phylogenetic lineages recognized by Guo *et al*.^12^. The red labels on the nodes of the phylogram indicate the new lineage (lineage 8) recognized in this research. The pink line represents the singleton. The values of the bootstrap frequencies of ML (BP > 70%) and posterior probability (PP > 0.90) are shown above the nodes. Armillaria isolates generated from this research are presented as M followed by a number.

In this study, we obtained a total of 47 *Armillaria* isolates with which *P. umbellatus* associated. The ITS, β-tubulin, EF-1α and three-locus matrices, derived from ML and BRC analyses yielded similar topologies. The three-locus matrix phylogenetic tree generated from ML and BRC analyses is shown in Fig. 1. The phylogenetic trees generated from ML analyses of ITS, β-tubulin, and EF-1α matrices are shown in Supplementary Information, Figure S1. Among the four matrices, ITS phylogeny (Supplementary Information, Figure S1C) and three-locus phylogeny (Fig. 1) present the lowest and the best branch resolution and support, respectively. Only a few branches were supported by bootstrap and posterior probabilities for the ITS phylogeny. The best branch resolution and support was obtained for the tree generated from three-locus phylogeny. From the three-locus phylogeny, Guo *et al*.^12^ revealed that there were at least 15 phylogenetic lineages of *Armillaria* in China. Our results support the 15 phylogenetic lineages. We further delimit an uncertain phylogenetic lineage that had been identified in a previous study, i.e. lineage 8. Lineage 8 is composed of two members, M20 (generated in this research) and a reported Chinese biological species (HKAS86607, CBS J), and was strongly supported by ML-BP (90%) and BRC-PP (0.97) in the three-locus phylogenetic tree. Lineage 8 was also strongly supported by ML-BP (99%) in the β-tubulin phylogenetic tree (Supplementary Information, Figure S1A).

**Figure 2:**
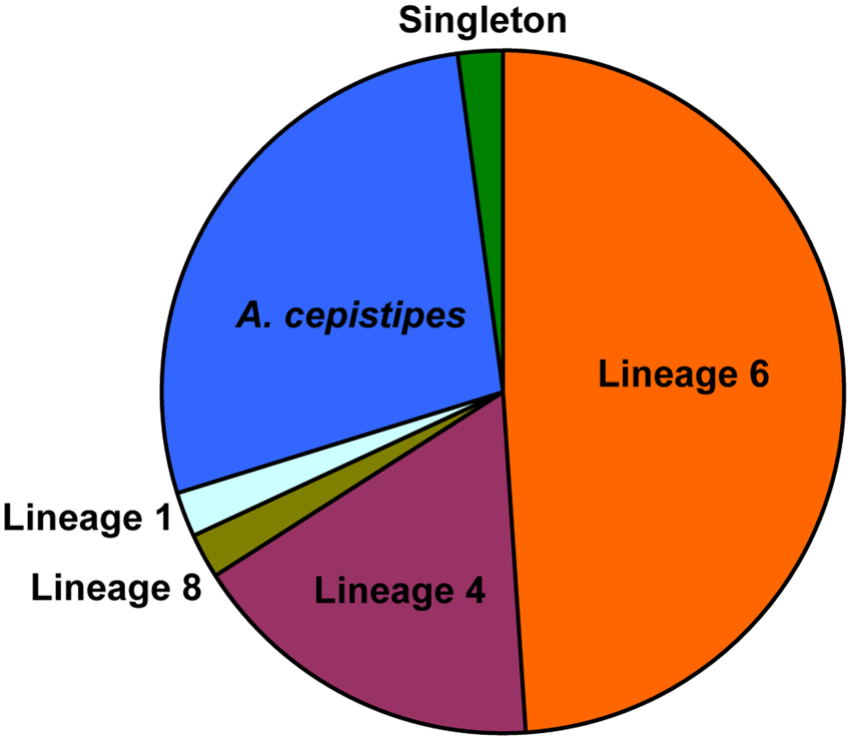
Relative abundance of the phylogenetic lineages of the forty seven *Armillaria* isolates.

The *Armillaria* isolates associated with *P. umbellatus* showed a high diversity and belonged to five independent phylogenetic lineages, including lineage 6, lineage 4, lineage 8, lineage 1, and *A. cepistipes*. Twenty-three *Armillaria* isolates, i.e., almost half of the total isolates, belonged to lineage 6. Thirteen isolates belonged to *A. cepistipes*. Eight isolates belonged to lineage 4. Lineage 1 and lineage 8 each include 1 isolate, respectively. Relative abundances of phylogenetic lineages of *Armillaria* isolates are shown in Fig. 2. One singleton (M3) showed relatively long branches compared with its sister group (Fig. 1). At present, the M3 strain is considered to be genetically divergent from its sisters.

Some isolates present evident geographic characteristics. Isolates from Shanxi, Gansu, Henan and Hebei provinces were all identified with lineage 6. Isolates from Northeast China, such as Jilin and Heilongjiang, belonged to *A. cepistipes*, except for isolate M47, which belonged to lineage 6. Isolates from Southwest China (Yunnan and Tibet) belonged to lineage 4. Among the 11 provinces sampled for *P. umbellatus* collected from, members of lineage 6 were found in 7 provinces (Shanxi, Shannxi, Gansu, Henan, Hebei, Sichuan and Jilin).

### Phylogenetic network analyses of *P. umbellatus*-*Armillaria* associations

When we examined the phylogenetic distance of the *Armillaria* strains associated with each of the *P. umbellatus* samples, the simple Mantel test showed that the phylogenetic distance of *P. umbellatus* and *Armillaria* strains were positively and significantly correlated (r = 0.4787, p < 0.01). This means that phylogenetically related *P. umbellatus* populations tend to interact with *Armillaria* species that are closely related.

**Figure 3:**
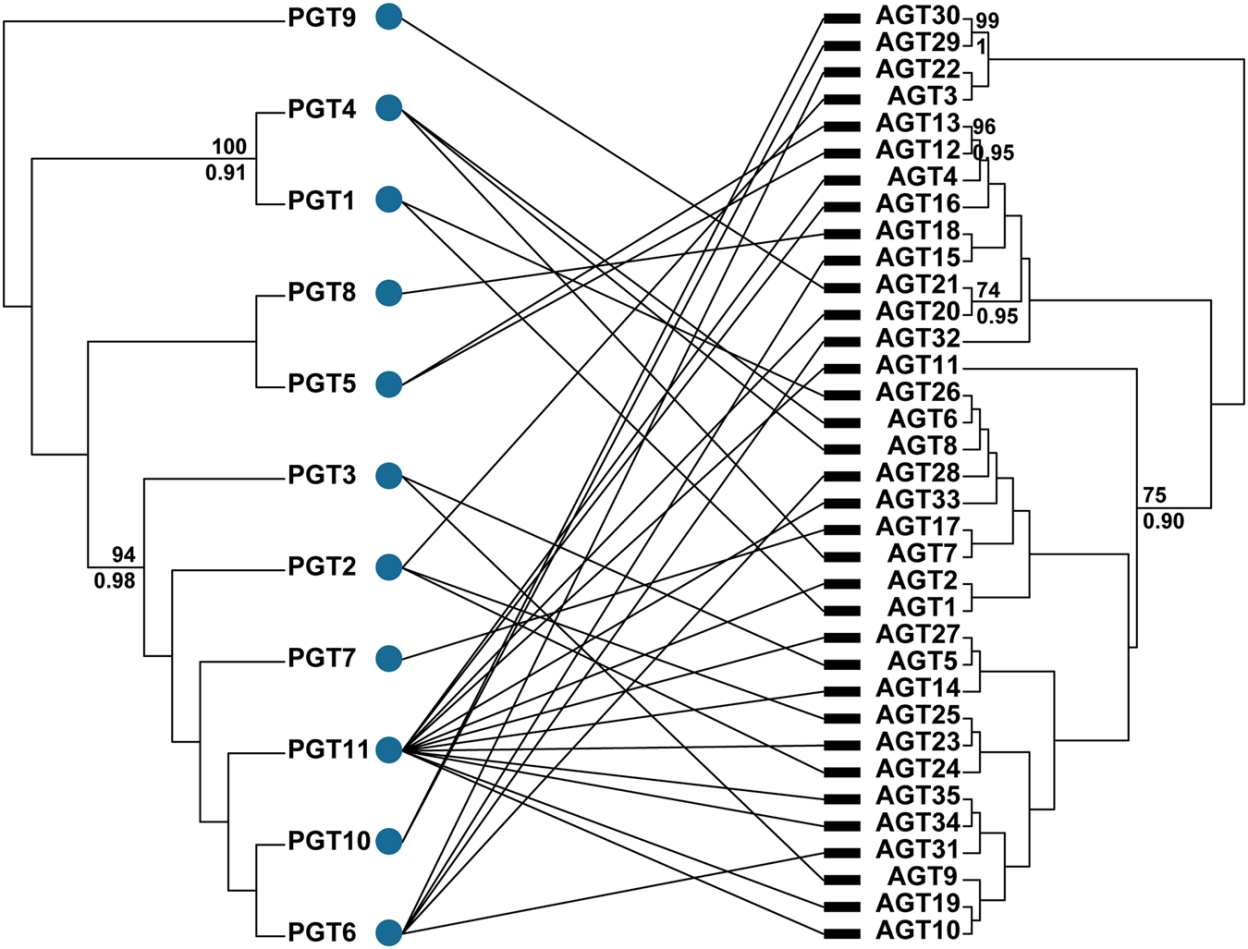
Network of *P. umbellatus*-*Armillaria* interactions. Lines represent pairwise interactions. Maximum clade credibility trees from Bayesian relaxed clock analyses are shown for *P. umbellatus* and *Armillaria* isolates. Branch support values above the branches show maximum likelihood nonparametric bootstrap percentages. Support values below branches are posterior probabilities. PGT, *P. umbellatus* genotype; AGT, *Armillaria* genotype.

To further understand the phylogenetic influence on the *P. umbellatus*-*Armillaria* associations, we incorporated the identity of the interacting taxa in the network (Fig. 3) and measured a moderate but significant phylogenetic signal on the *Armillaria* phylogeny, both when considering the ML phylogeny (*d*_A_ = 0.3522; 95% CI 0.1496–0.5327) and when considering the BRC phylogeny (*d*_A_ = 0.2109; 95% CI 0.0508–0.3574). The phylogenetic signal of the *P. umbellatus* was close to zero and not significant: for the ML tree, *d*_P_ < 0.0001 (95% CI 0–0.0100), and for BRC tree, *d*_P_ < 0.0001 (95% CI 0–0.0047). The overall strength of the phylogenetic signal for the linear model fitted to the actual data (MSE_*d*_ = 0.1282 and MSE_*d*_ = 0.1382 for the ML and BRC tree sets, respectively) was closer to that found under the assumption of no phylogenetic covariances (MSE_*star*_ = 0.0829) than for the assumption of maximum phylogenetic signal (MSE_*b*_ = 0.4655 and MSE_*b*_ = 0.6035) for the ML and BRC tree sets, respectively). These results suggest that only phylogenetic relationships among the *Armillaria* species and not among the *P. umbellatus* impose structure on the interaction matrix. However, the overall phylogenetic signal is weak.

## Discussion

A subset of species in the *Armillaria* genus are important plant pathogens and can cause serious root diseases in diverse trees and woody plants. Interestingly, some species of *Armillaria* are also well known as symbionts of *Gastrodia elata* Bl. (a myco-heterotrophic orchid used in traditional Chinese herbal medicine)^17^ and *P. umbellatus*. Taxonomic classification of *Armillaria* is complicated by high intraspecific diversification and the lack of sexual stages in many species. Despite early mating tests^18^ as well as studies that have utilized recently developed DNA data analysis^19, 20^ and mutilocus approaches^21^, there are still some ambiguous groups and unnamed biological species. In China, 16 CBS of *Armillaria* were identified by mating tests. However, due to the limits of mating tests, these CBS still need further verification via by modern molecular approaches. Coetzee *et al*.^11^ elucidated the four main phylogenetic groups of *Armillaria* in China. Subsequently, Guo *et al*.^12^ effectively established the link between the CBS and the phylogenetic lineages and identified at least 15 phylogenetic lineages in China. These works gradually clarified the taxonomy of *Armillaria* in China.

*P. umbellatus* sclerotial growths require the *Armillaria* rhizomorph to supply nutrition. To date, l little is known about the exact *Armillaria* species associated with *P. umbellatus*. In this study, we determined that all the Armillaria isolates belong to the 4 phylogneetic lineages recognized by Guo *et al*.^12^, except for two isolates, M20 and M3. We then further delimited an uncertain phylogenetic lineage found in a previous study (lineage 8), which was composed of one isolate (M20) generated in this research and another singleton (HKAS86607 CBS J)^12^ supported by high ML and RBC bootstrap. Ultimately, only one singleton (M3) did not belong to any lineages. Its classification requires further study. Lineage 6 included almost half of the total number of isolates. This lineage was defined as a new phylogenetic lineage in Guo *et al*.^12^. This lineage is represented in most of the samples from China previously considered as *A. gallica*, which was strongly divergent from European *A. gallica*. However, we also found evident divergence in lineage 6 which formed two subgroups in the β-tubulin phylogeny (Supplementary Information, Figure S1A). In contrast, ML bootstrap for this lineage was low in the EF-1α phylogeny (<70%) (Supplementary Information, Figure S1B). Twelve isolates in this study together with samples previously considered as *A. gallica* formed one subgroup, while ten isolates in this research with *A. gallica* (HKAS85517) and a CBS B (HKA86573) formed another subgroup. A probable reason for this result is that *A. gallica* has clear intraspecific differentiation and may be in the process of allopatric speciation^22^.

From this research, we have shown that *P. umbellatus* associates with diverse *Armillaria* partners. Previously published articles named the *Armillaria* species with which *P. umbellatus* associated as *A. mellea* or *Armillaria* spp. However, there has been no evidence to date to suggest that *A. mellea* is the fungal partner of *P. umbellatus*. Additionally, the *Armillaria* species used in the cultivation of *P. umbellatus* have not been identified. This study also found that the *Armillaria* isolates present certain geographic characteristics. For example, *Armillaria* isolates from Northeast China are mainly *A. cepistipes*, while isolates from Southwest China mainly belong to lineage 4. Although present results may not totally reflect the true *Armillaria* communities in some provinces due to small sample sizes, it is clear that different *Armillaria* isolates must be selected when used in cultivation of *P. umbellatus* in different regions. Inappropriate *Armillaria* isolates may lead to unstable yield and production efficiency.

The evolution of traits involved in ecological interactions such as predator-prey, host-parasite, and plant-pollinator interactions, are likely to be shaped by the phylogenetic history of both parties. In the *P. umbellatus*-*Armillaria* interactions, the phylogenetic distance of *P. umbellatus* and *Armillaria* strains were positively and significantly correlated. This means that phylogenetically related *P. umbellatus* populations tend to interact with a closely related *Armillaria* species. However, the *P. umbellatus* phylogeny does not show a significant phylogenetic signal on the interaction with their associated *Armillaria* species, but the *Armillaria* exhibits a significant phylogenetic signal on the interaction. Such asymmetric patterns have also been reported in other systems, e.g. orchid mycorrhizal symbiosis^23, 24^ and ectomycorrhizal symbiosis^25^. Additionally, within tropical and parasitic networks, interaction conservatism is often stronger for resources than for consumers. This means that related prey species tend to share more consumers than related consumers share prey species^26–30^. In the *P. umbellatus*-*Armillaria* interaction, sclerotia of *P. umellatus* digested the penetrated rhizomorph of *Armillaria* to meet their nutrition demands. However, the *Armillaria* are not dependent on *P. umbellatus* for their reproduction and dispersal and can survive as either saprophytes or parasites. Their distribution is independent of *P. umbellatus*. *Armillaria* species are a major component of the mycobiota of many forest ecosystems, however, the origin and diversification of this genus is complicated and poorly known. Some of the *Armillaria* species present considerable intraspecific genetic differentiation, and are in the process of allopatric speciation^22, 31, 32^. In addition to the intraspecific genetic diversity of *Armillaria* species, *P. umbellatus* also contains levels of intraspecific genetic diversity^16^. It is unlikely that *Armillaria* species have evolved substantially in response to the *P. umbellatus*, which may explain the asymmetric relationship of *P. umbellatus-Armillaria* associations.

## Methods

### Collection of *P. umbellatus* sclerotial samples

**Figure 4:**
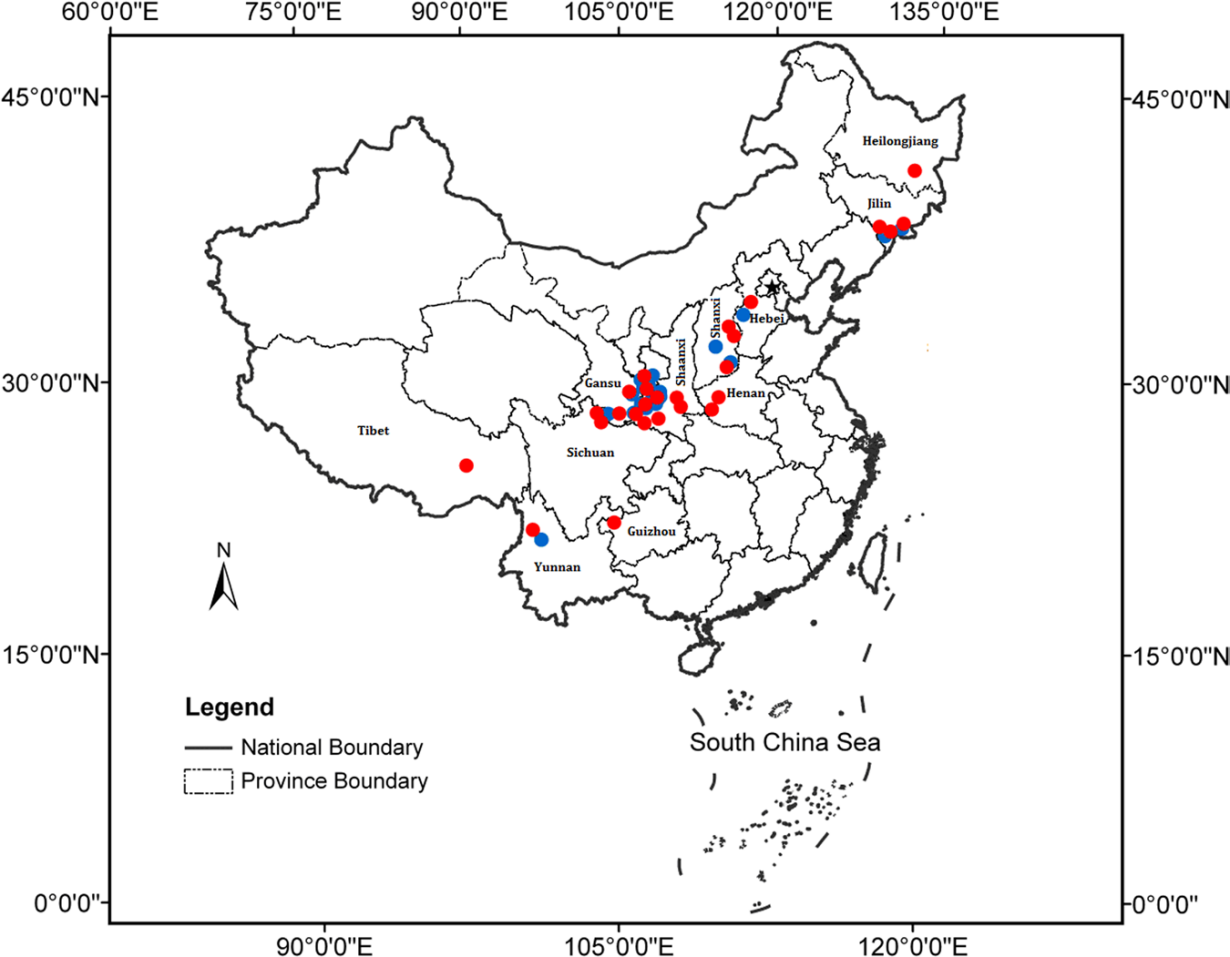
Map of China showing successful sampling sites of *P. umbellatus*. Red circles: the *P. umbellatus* samples from which Armillaria were isolated; Blue circles: the *P. umbellatus* samples from which *Armillaria* were not successfully isolated. The map was generated using ArcGIS 9.3 (ESRI, Redlands, CA, USA; http://www.esri.com).

We collected 57 wild sclerotial samples of *P. umbellatus* from the following eleven provinces of China: Heilongjiang, Jilin, Shanxi, Shaanxi, Henan, Yunnan, Gansu, Sichuan, Tibet, Guizhou and Hebei. (Fig. 4). Details regarding the samples are shown in Supplementary Information, Table S1. For each sample, at least 12 sclerotia from different individuals growing 30–50 m apart were chosen. In total, more than 684 individual sclerotia were collected. Once the fresh sclerotia were collected, they were delivered to the lab within two to three days for further processing. Some sclerotia from each sample were also numbered and allowed to air-dry at room temperature. These sclerotia were then deposited in the herbarium of the Institute of Medicinal Plant Development, Chinese Academy of Medical Sciences.

### Isolation of *Armillaria*

**Figure 5:**
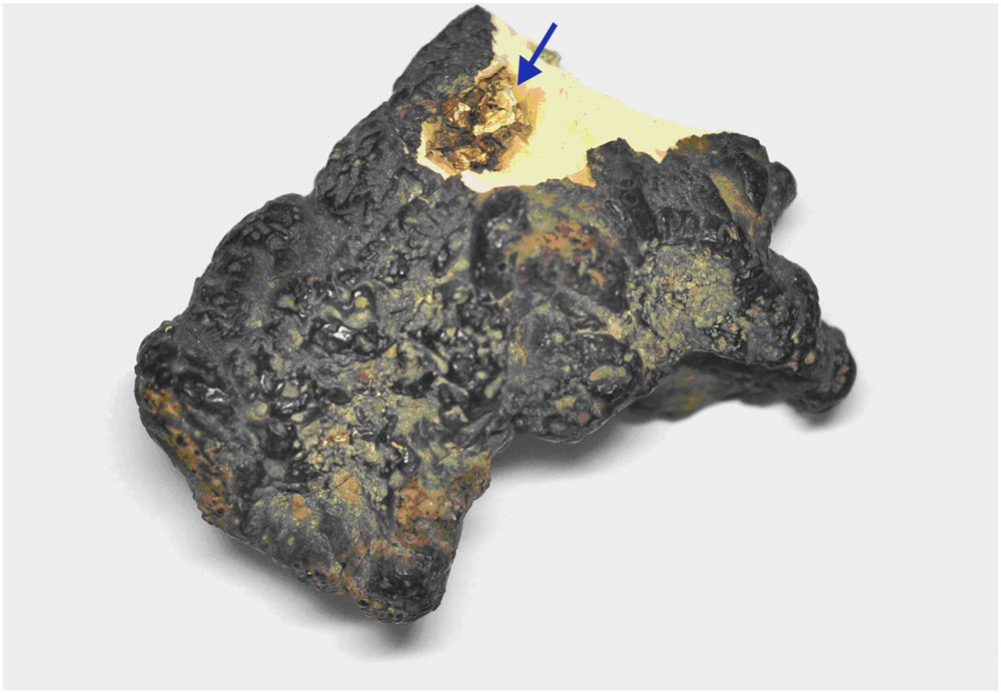
Sclerotia of *P. umbellatus* bisected from the penetration site of *Armillaira* rhizomorph on the sclerotial surface. The arrow shows a cavity beneath the surface which contains the penetrated *Armillaira* rhizomorph.

The sclerotia of *P. umbellatus* were washed thoroughly in running tap water for 10 min and were surface sterilized via submerion in 75% ethanol for 1 min, a solution of 3.5% (v/v) Chlorox for 2 min and 75% ethanol for 30 s. The surface sterilized sclerotia were washed with sterile distilled water three times and blotted with sterile absorbent paper. Ten individual sclerotia from each sample were used for *Armillaria* isolation. In order to obtain the *Armillaria* isolates which actually infected sclerotia of *P. umbellatus*, we only isolated the *Armillaria* from the infection cavity inside the sclerotia. To accomplish this, the surface sterilized sclerotia were bisected from the evident penetration site of the *Armillaria* rhizomorph on the sclerotial surface. Once the sclerotia were cut from the correct site, a cavity where *Armillaria* had colonized can be seen (Fig. 5).

The residual *Armillaria* rhizomorph in the cavity was extracted and placed on potato dextrose agar (PDA) medium plates amended with streptomycin to suppress the growth of bacteria. As the *Armillaria* rhizomorph in the cavity were in different digestion stage, only the newly penetrated and undigested rhizomorph could be selected. This resulted in a low successful isolation rate of *Armillaria* strain. The selected rhizomorph of *Armillaria* were extracted and cultured. Plates were incubated at 23 °C in the dark. The growing tips of the *Armillaria* rhizomorph were transferred to new plates. All isolates were numbered and kept for further identification.

### DNA extraction

An *Armillaria* rhizomorph was extracted out from each plate using sterile forceps. The rhizomorph surface attached to the media were removed carefully using a dissecting needle. For the *P. umbellatus* sclerotial DNA extraction, the sclerotia were then cut in half and 100 mg of medullar tissue was removed. Both sclerotia and rhizomorph samples were ground with a mortar and pestle in liquid nitrogen. Genomic DNA was extracted using the E.Z.N.A. ^TM^ Fungal DNA kit (Omega) following the manufacturer’s instructions.

### PCR amplication and sequencing

The ITS, β-tubulin and elongation factor-1 alpha (EF-1α) have been used to infer phylogenetic relationships for various species of *Armillaria* ^33–35^. In this study of *Armillaria* fungal *strains*, we amplified the ITS1-5.8 S rDNA-ITS2 using the universal primer pair ITS1 and ITS4^36^, the EF-1α using pair EF595F/EF1160R^37^, and the β-tubulin using primer pair TubF/TubR^34^. For *P. umbellatus*, we amplified the ITS1-5.8 S rDNA-ITS2 using the universal primer pair ITS1 and ITS4^36^. PCR amplification was performed in a 25 μL reaction volume containing approximately 20 ng of DNA, 1 μL of each primer, and 12.5 μL of PCR master mix (Aidlab Biotech Co., Ltd., Beijing, China). All PCR reactions were carried out on a BIO-RAD T100 Thermal Cycler under the following reaction conditions. ITS followed predenaturation at 95 °C for 10 min, followed by 36 cycles of denaturation at 94 °C for 15 s, annealing at 53 °C for 30 s, and elongation at 72 °C for 2 s. A final elongation at 72 °C for 7 min was included after the cycles. β-tubulin followed predenaturation at 94 °C for 3 min, followed by 35 cycles of denaturation at 95 °C for 40 s, annealing at 53 °C for 40 s, and elongation at 72 °C for 90 s. EF-1α followed predenaturation at 95 °C for 10 min, followed by 35 cycles of denaturation at 94 °C for 15 s, annealing at 56 °C for 30 s, and elongation at 72 °C for 30 s. A final elongation at 72 °C for 30 s was included after the cycles. The PCR products were separated on a 1% (w/v) agarose gel and the bands were visualized under UV illumination. For most of the samples, PCR amplification yielded a single strong band. PCR products that could not be sequenced successfully were cloned into a Trans 5α vector (TransGen Beijing, China) and then sequenced with universal primers M13F /M13R. The contiguous sequences were assembled with SeqMan (DNASTAR Inc., USA). The sequences for ITS, β-tubulin and EF-1α of *Armillaria* isolates and ITS sequences of *P. umbellatus* were deposited in GenBank (accession numbers KY389147 - KY389193, KY389267 - KY389313, KY389220 - KY389266, and KY389194 - KY389219).

### Sequence alignment and phylogenetic analyses

In order to understand the phylogenetic relationship between *Armillaria* isolates generated in this research and the phylogenetic lineages of *Armillaria* in China recognized by Guo *et al*.^12^, four matrices were compiled in this research, i.e. ITS, β-tubulin, EF-1α and three-locus matrices. We downloaded the sequences of ITS, β-tubulin and EF-1α published by Guo *et al*.^12^ from GenBank. All ITS, β-tubulin and EF-1α, and reference sequences were aligned with Clustal X version 2.0^38^, respectively, and ambiguous regions in both sides of each region were excluded.

The best-fitted substitution model for each matrix was determined via jModelTest 2^39^ based on the Akaike Information Criterion (AIC). TN93+G+I and T92+G were selected as the best models for the three-locus and ITS matrices, respectively. K2+G was selected as the best model for the EF1-α and the β-tubulin matrix, respectively. Maximum likelihood (ML) bootstrap analyses were conducted for the four matrices. ML phylogeny was constructed with RAxML 7.0.4^40^. Clade support was estimated with RAxML via nonparametric bootstrap analysis on 1000 pseudo-replicate data sets. In addition to the ML trees, we constructed ultrametric trees with a BRC analysis using BEAST 1.5.4^41^. The uncorrelated lognormal clock model^42^ was selected and a pro forma calibration point was enforced: the root height was fixed at 1.0. Posterior distributions of parameters were approximated using two independent Markov chain Monte Carlo analyses of 2.0 × 10^7^generations followed by a discarded burn-in of 2.0 × 10^6^ generations (10%).

### Data analyses

In order to test whether phylogenetic relatedness of *P. umbellatus* samples correlates with phylogenetic relatedness of *Armillaria* species, a simple Mantel test implemented in ZT 1.1^43^ was used to compare phylogenetic distance matrices of *P. umbellatus* with phylogenetic distance matrices of associated *Armillaria* strains. The phylogenetic distance for both *P. umbellatus* and *Armillaria* strains was calculated using the ‘distance’ option in Geneious 8.1.6 (http://www.geneious.com) based on the highest likelihood tree from the ML analysis. The simple Mantel test was run with 10000 randomizations.

Besides the phylogenetic relatedness of *P. umbellatus*-*Armillaria* associations, we further evaluated the strength of phylogenetic signal of the two phylogenies on the *P. umbellatus*–*Armillaria* interactions via using a linear model approach that fits the phylogenetic variance–covariance matrix to the plant–fungi interaction matrix^26^. ITS sequences of both *P. umbellatus* and *Armillaria* isolates were used. Prior to the analysis, we first analyzed the pairwise distances of all the *P. umbellatus* samples and *Armillaria* isolates, respectively, we then treated the pairwise distances equal to zero as one genotype and the pairwise distances >0 as different genotypes. Finally, the 26 *P. umbellatus* samples were classed into 11 genotypes, and the 47 *Armillaria* isolates were classed into 35 genotypes. We then generated a *P. umbellatus-Armillaria* interaction matrix composed of 0/1 (present/absent) data. Because measurements of phylogenetic signal are based on evolutionary rates (branch lengths) estimated by phylogenetic inference, we examined phylogenetic signal on ML trees, where branch lengths are estimated without a molecular clock assumption and represent genetic distance, and Bayesian relaxed clock (BRC) trees, where branch lengths are estimated under a relaxed molecular clock assumption and represent time. The ITS sequences of 11 *P.umbellatus* genotypes and 35 *Armillaria* genotypes were aligned with Clustal X version 2.0^38^, respectively. K2+G and T92+G models of evolution were identified as the best-fit model for the *P. umbellatus* and *Armillaria* data sets, respectively, using AIC implemented in jModelTest 2^39^.

We applied the phyogenetic bipartite linear model of Ives and Gofray^26^. The structure of the association matrix is decomposed into a phylogenetically corrected mean association strength and a vector of residuals depending on the phylogenies via an estimated general least square (EGLS) analysis. The reference evolution model used to calculate the phylogenetic structure is the Ornstein–Uhlenbeck (OU) process, which can incorporate stabilizing selection^44^. We calculated the independent phylogenetic signals of the *P. umbellatus* (*d*_P_) and *Armillaria* (*d*_A_) phylogenies on the interaction matrix (association present/absent) and the strength of the signal of both phylogenies combined (MSE*d*). MSEd was compared with MSE values for a model that assumes no phylogenetic structure (MSE*star*) and a Brownian evolution model (MSE*b*). The model minimizing the mean squared error was considered the best fit. Bipartite linear models were performed using the pblm function in the *picante* R package^45^ and were carried out on the ML and BRC results for *P. umbellatus*–*Armillaria* phylogeny sets. Statistical significance of the *d* values was estimated by calculating 95% bootstrap confidence intervals on 100 replicates.

### Data availability

All data generated during this study are included in this published article (and its Supplementary Information files).

## Electronic supplementary material

Table S1

Figure S1
