# Supplementary material for: Phylogenetic constrains on Polyporus umbellatus-Armillaria associations
Source: Sci Rep. 2017 Jun 26;7:4226. doi: 10.1038/s41598-017-04578-9 (PMC5484660; doi:10.1038/s41598-017-04578-9)
Supplement: Supplementary file 1 — Table S1 [file 41598_2017_4578_MOESM1_ESM.doc]

**Supplementary material_Table S1**

Article title: Phylogenetic constrains on *Polyporus umbellatus* - *Armillaria* associations

Journal name: Scientific Reports

Author names and affiliation: Xiaoke Xing, Jinxin Men, Shunxing Guo. *Institute of Medicinal Plant Development, Chinese Academy of Medical Sciences and Peking Union Medical College, Beijing 100193, China.*

E-mail address of the corresponding author: xkxing2009@hotmail.com; sxguo@implad.ac.cn

**Table S1** Number of the *P. umbellatus* samples, the isolated *Armillaria* strains and sampling sites.

| Samples of *P. umbellatus* | *Armillaria* isolates | Sampling sites | Latitude-longitude |
| --- | --- | --- | --- |
| Z1 | M1 | Hailin, Heilongjiang | N44°36′, E129°23′ |
| Z3 | M3 | Hanzhong, Shaanxi | N33°04′, E107°01′ |
| Z4 | M4 | Jincheng, Shanxi | N35°47′, E113°17′ |
| Z5 | M5 | Yangquan, Shanxi | N35°47′, E113°17′ |
| Z6 | M6-1, M6-2 | Yangquan, Shanxi | N35°47′, E113°17′ |
| Z7 | M7-1, M7-2, M7-3, M7-4 | Baishan, Jilin | N41°59′, E127°13 |
| Z8 | M8-1, M8-2, M8-2 | Xixia, Henan | N33°37′, E111°44′ |
| Z11 | M11-1, M11-2, M11-3, M11-4, M11-5, M11-6, M11-7 | Nujiang, Yunnan | N26°27′, E99°25′ |
| Z18 | M18-1, M18-2 | Longnan, Gansu | N33°42′, E106°25′ |
| Z19 | M19 | Jiuzhaigou, Sichuan | N33°16′, E103°55 |
| Z20 | M20 | Jiuzhaigou, Sichuan | N33°16′, E103°55 |
| Z21 | M21 | Baoji, Shaanxi | N34°54′, E106°52′ |
| Z22 | M22 | Linzhi, Tibet | N29°52′, E95°46′ |
| Z23 | M23-1, M23-2 | Songxian, Henan | N33°42′, E111°55′ |
| Z24 | M24 | Hezhang, Guizhou | N27°07′, E104°48′ |
| Z25 | M25 | Mianxian, Shaanxi | N33°09′, E106°30′ |
| Z26 | M26 | Laiyuan, Hebei | N39°33′, E114°41′ |
| Z27 | M27-1, M27-2 | Lueliang, Shanxi | N37°31′, E112°04′ |
| Z28 | M28-1, M28-2 | Songjianghe, Jilin | N42°11′, E127°29′ |
| Z29 | M29-1, M29-2 | Zhashui, Shaanxi | N33°50′, E109°16′ |
| Z31 | M31-1, M31-2 | Zhashui, Shaanxi | N33°50′, E109°16′ |
| Z46 | M46-1, M46-2 | Fengxian, Shaanxi | N33°42′, E106°32′ |
| Z47 | M47 | Liuhe, Jilin | N42°13′, E126°0′ |
| Z49 | M49-1, M49-2 | Pingliang, Gansu | N35°33′, E106°40′ |
| Z50 | M50-1, M50-2 | Baoji, Shaanxi | N34°04′, E107°19′ |
| Z51 | M51 | Tianshui, Gansu | N35°13′, E106°39′ |
