# Supplementary material for: Phylogenetic constrains on Polyporus umbellatus-Armillaria associations
Source: Sci Rep. 2017 Jun 26;7:4226. doi: 10.1038/s41598-017-04578-9 (PMC5484660; doi:10.1038/s41598-017-04578-9)

**Supplementary material_Figure S1**

Article title: Phylogenetic constrains on *Polyporus umbellatus* - *Armillaria* associations

Journal name: Scientific Reports

Author names and affiliation: Xiaoke Xing, Jinxin Men, Shunxing Guo. *Institute of Medicinal Plant Development, Chinese Academy of Medical Sciences and Peking Union Medical College, Beijing 100193, China.*

E-mail address of the corresponding author: xkxing2009@hotmail.com; sxguo@implad.ac.cn

**Figure S1** Phylogenetic trees generated from the β-tubulin, EF1-α and ITS (C) dataset, respectively. The blue labels on the nodes of the phylogram indicate phylogenetic lineages recognized by Guo et al. (2016). The pink brackets indicate the singletons. The values of the bootstrap frequencies of ML (BP>70%) and posterior probability (PP>0.90) are shown above the nodes. *Armillaria* isolates generated from this research are presented as M followed by number. **A.** Phylogenetic trees generated from the β-tubulin dataset. Lineage 6 was divided into two lineages. One new lineage, that is lineage 8 recognized in this research was of high bootstrap support in the β-tubulin phylogeny. **B.** Phylogenetic tree generated from the EF1-α dataset. Five lineages (lineage 2,3,4,5,8) combined to one lineage in the EF1-α phylogeny. **C.** Phylogenetic tree generated from the ITS dataset. Only four lineages defined in the ITS phylogeny. Most of the lineages formed a lineage complex.

**A**


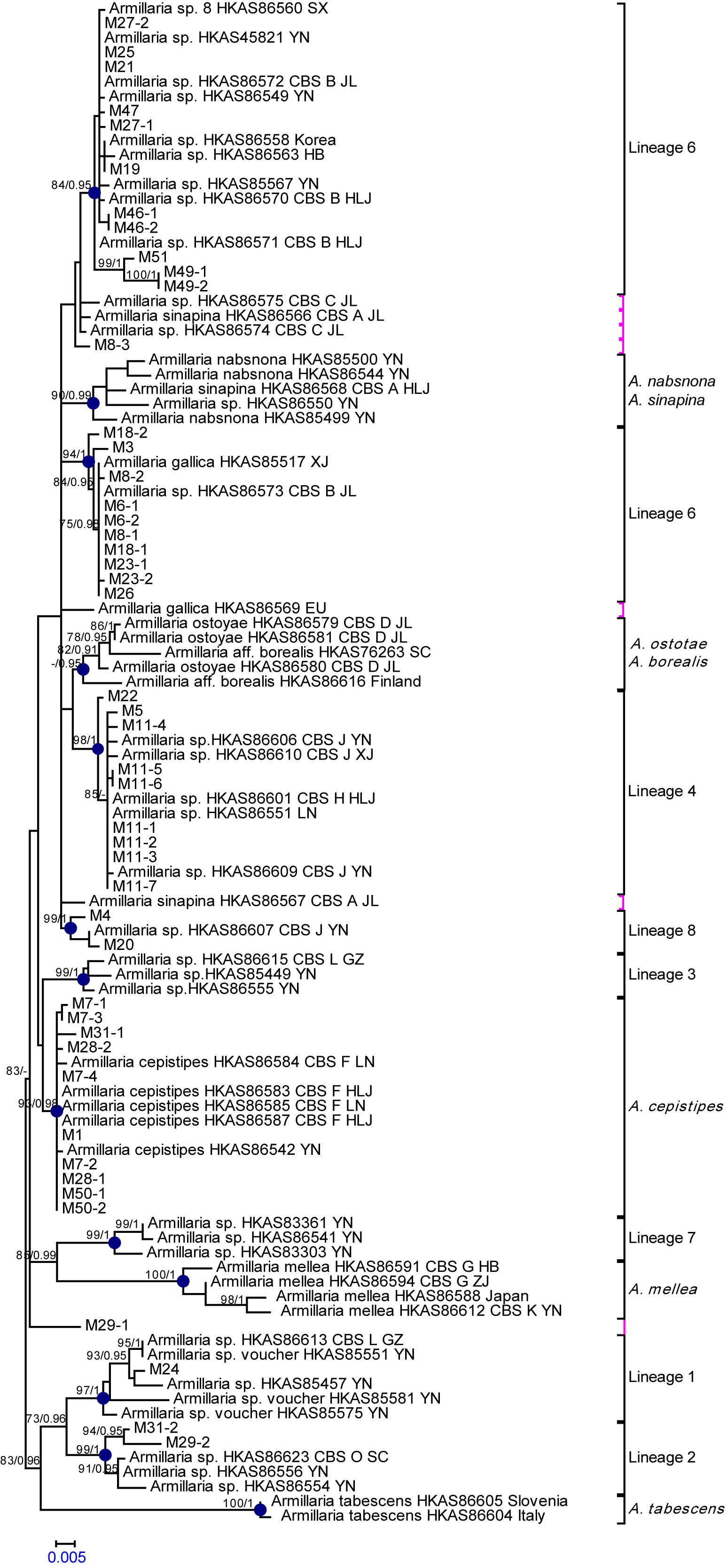


**B**

**
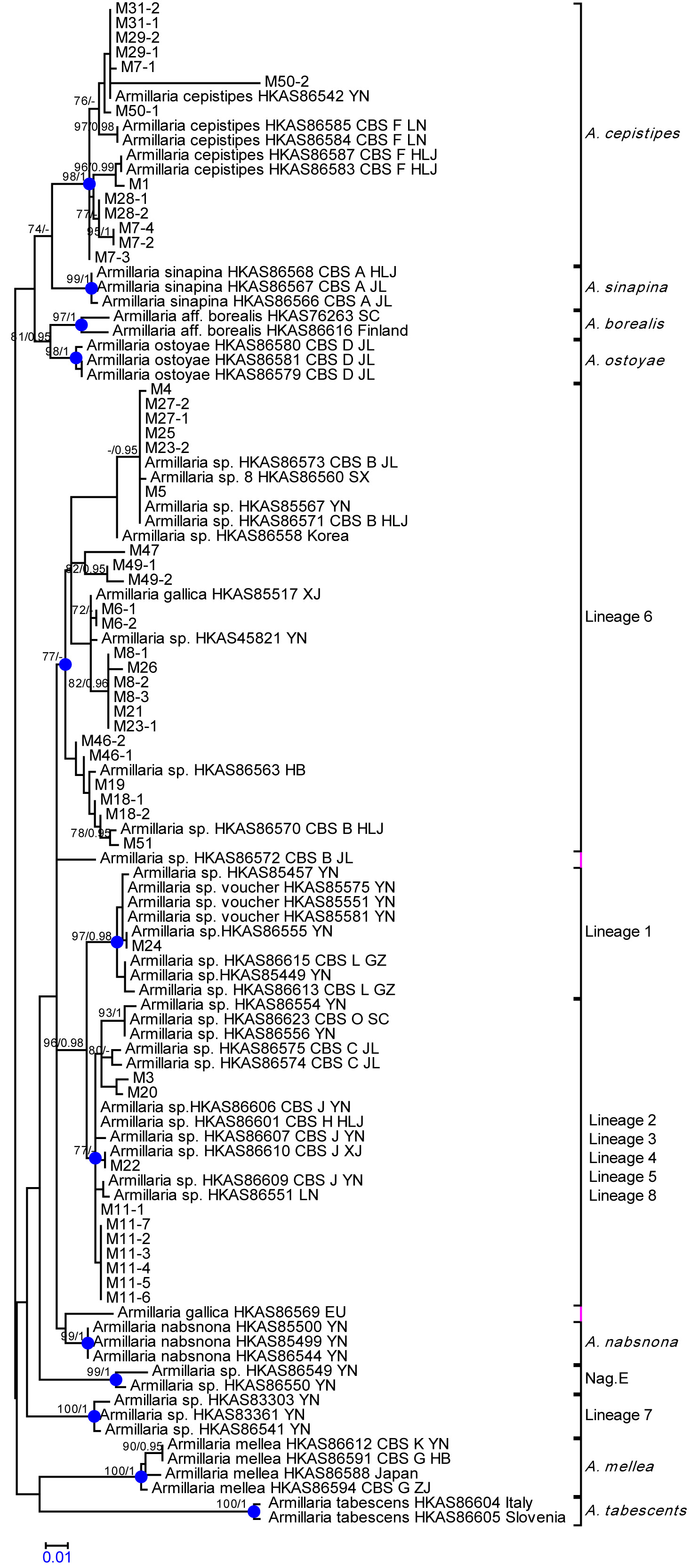
**

**C**


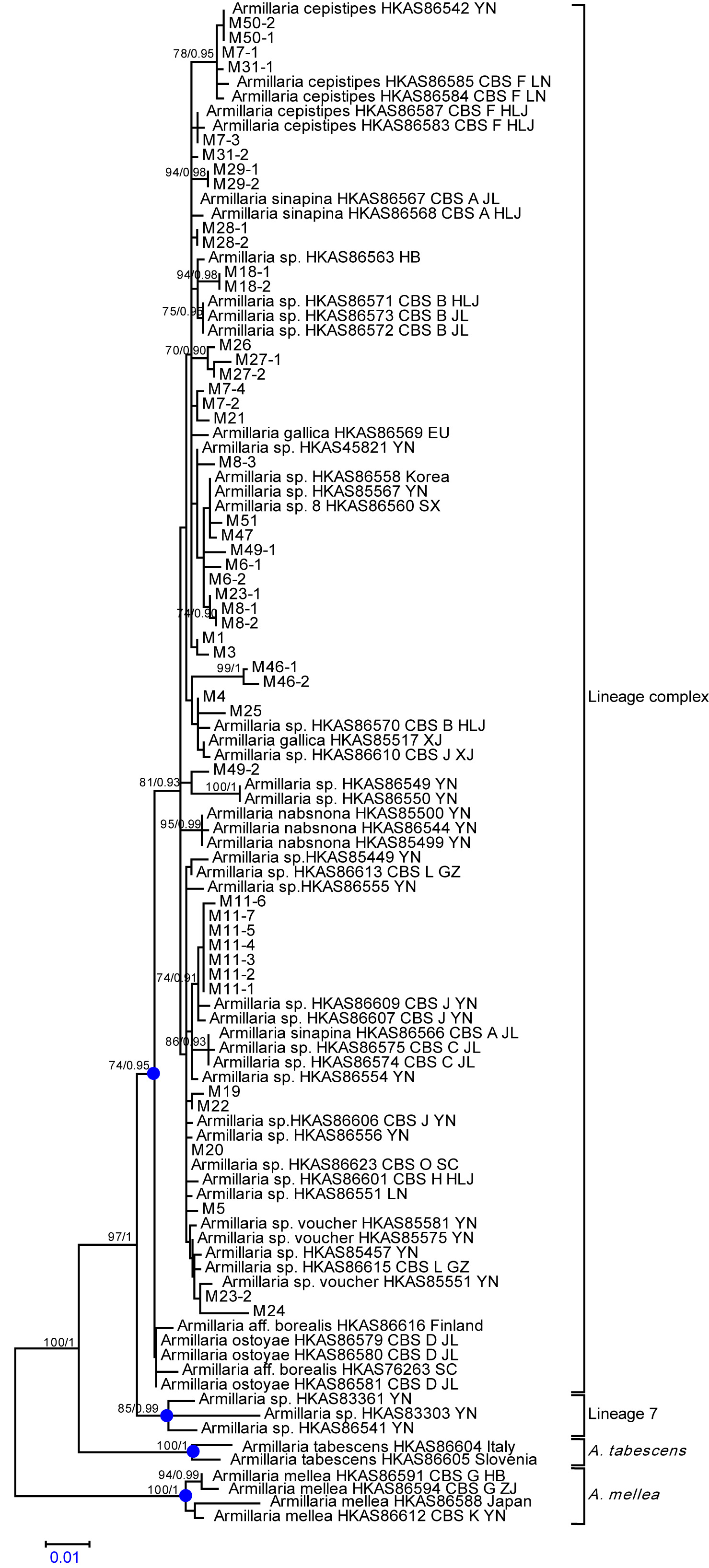

Supplement: Supplementary file 2 — Figure S1 [file 41598_2017_4578_MOESM2_ESM.doc]
